# Supplementary figures and images for: Zika virus E protein modulates functions of human brain microvascular endothelial cells and astrocytes: implications on blood-brain barrier properties
Source: Front Cell Neurosci. 2023 Jul 20;17:1173120. doi: 10.3389/fncel.2023.1173120 (PMC10399241; doi:10.3389/fncel.2023.1173120)

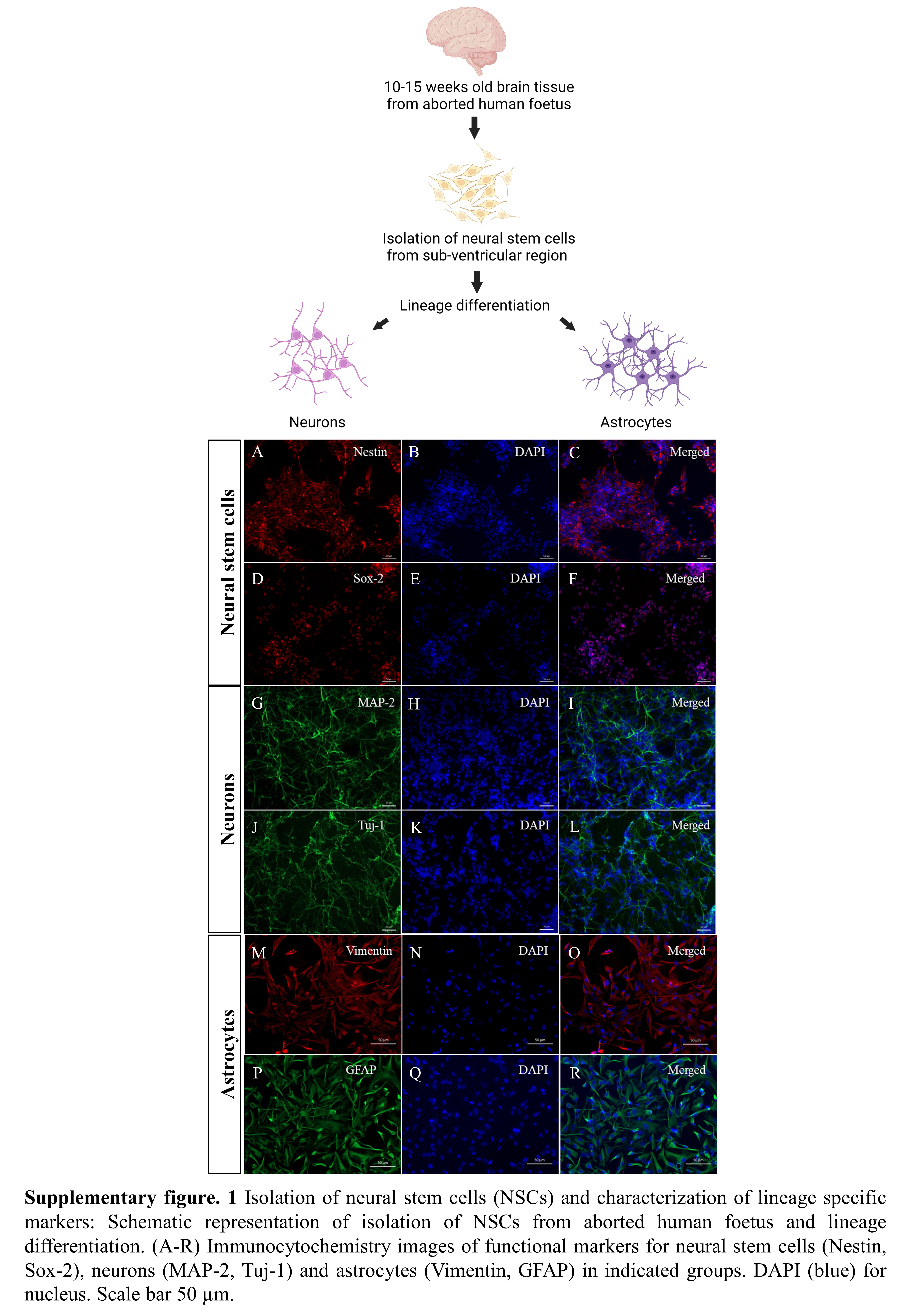

Supplement: Supplementary file 2 [file Image_1.JPEG]

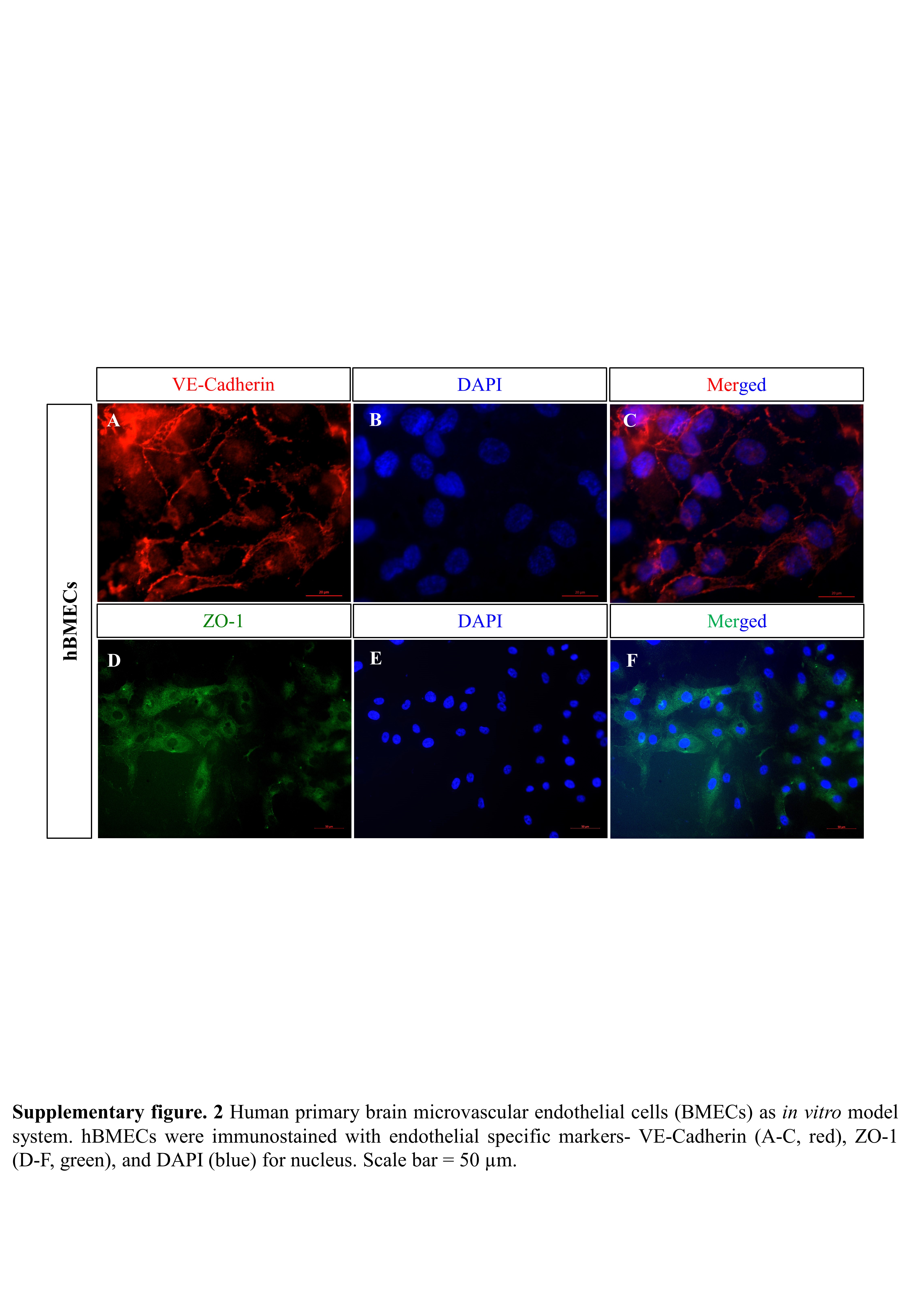

Supplement: Supplementary file 3 [file Image_2.JPEG]

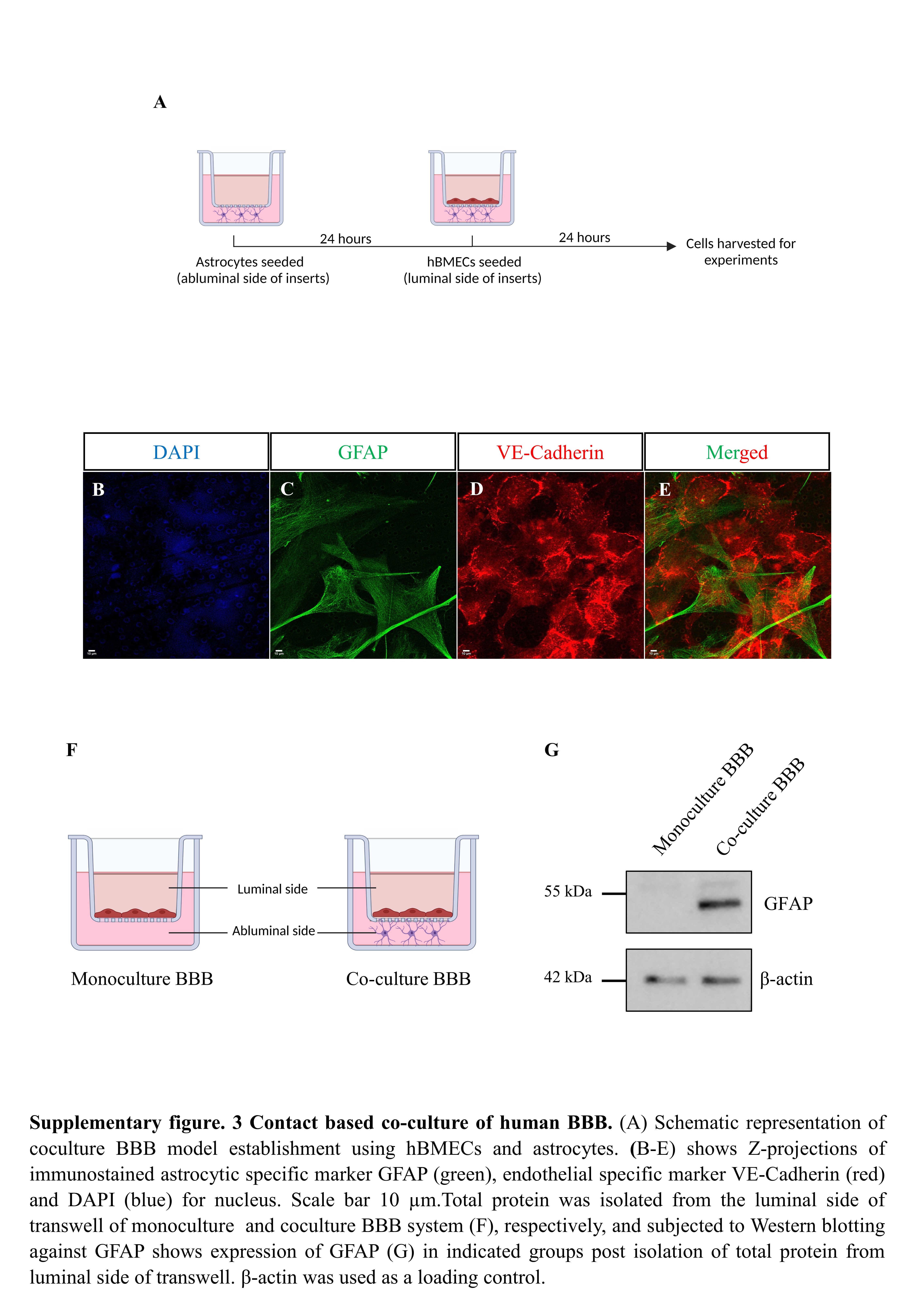

Supplement: Supplementary file 4 [file Image_3.JPEG]

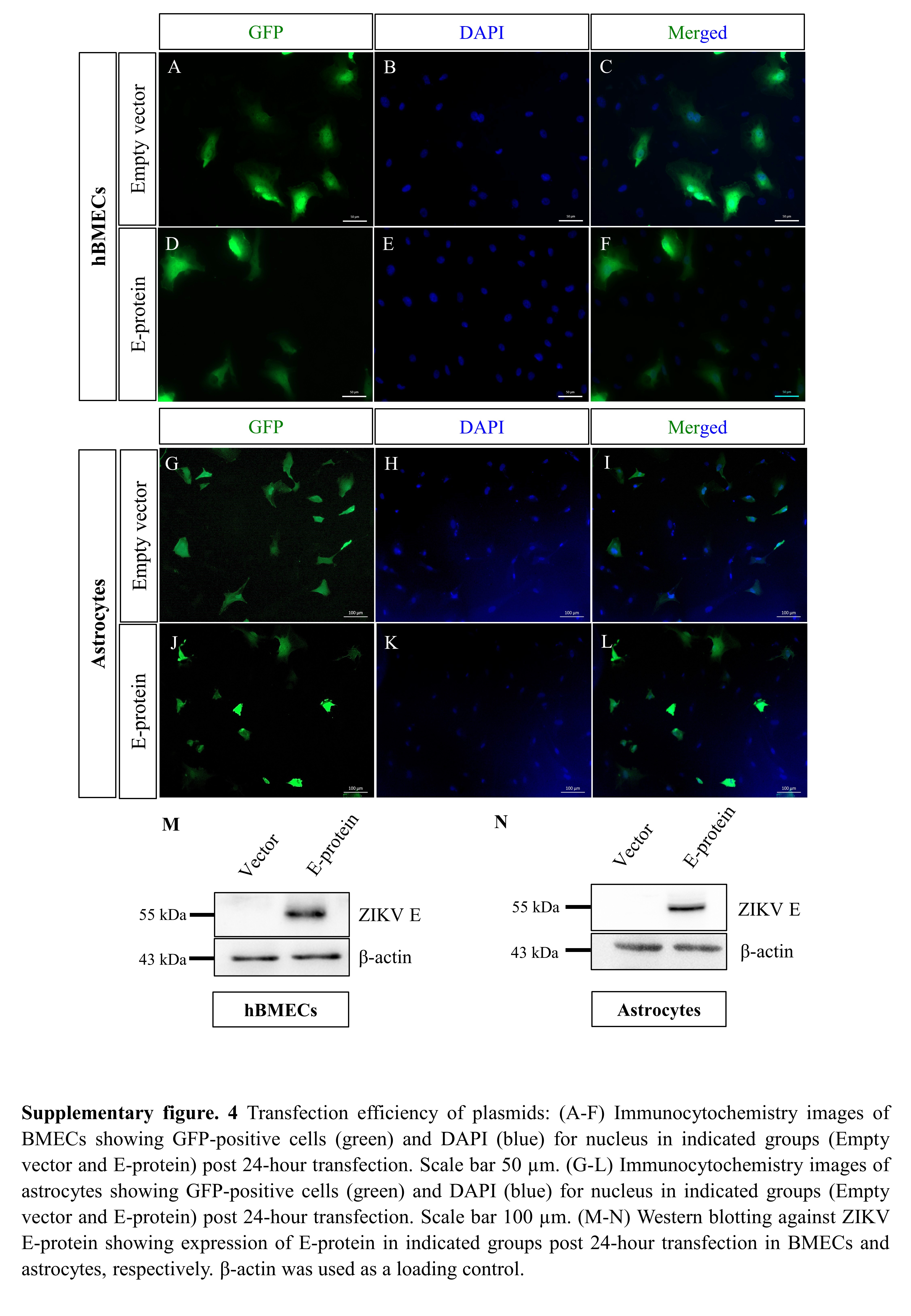

Supplement: Supplementary file 5 [file Image_4.JPEG]
